# Trypanosome infection rates in tsetse flies in the “silent” sleeping sickness focus of Bafia in the Centre Region in Cameroon

**DOI:** 10.1186/s13071-015-1156-z

**Published:** 2015-10-12

**Authors:** Gustave Simo, Pierre Fongho, Oumarou Farikou, Prosper Innocent Ndjeuto Ndjeuto-Tchouli, Judith Tchouomene-Labou, Flobert Njiokou, Tazoacha Asonganyi

**Affiliations:** Molecular Parasitology and Entomology Unit, Department of Biochemistry, Faculty of Science, University of Dschang, PO Box 67, Dschang, Cameroon; Faculty of Science, University of Yaoundé I, PO Box 812, Yaoundé, Cameroon; Ministry of Livestock, Fisheries and Animal Industries, Special Mission for TseTse Flies Eradication, PO Box 263, Ngaoundéré, Cameroon; Department of Geography, University of Ngaoundéré, Ngaoundéré, Cameroon; Faculty of Medicine and Biomedical Sciences, University of Yaoundé 1, Yaoundé, Cameroon

**Keywords:** Sleeping sickness, African animal trypanosomiasis, Trypanosomes, *Glossina palpalis palpalis*, Mid-guts

## Abstract

**Background:**

The Bafia sleeping sickness focus of Cameroon is considered as “silent” with no case reported for about 20 years despite medical surveys performed during the last decades. In this focus, all epidemiological factors that can contribute to trypanosomes transmission are present. To update our knowledge on the current risks of Human and Animal African trypanosomiases, different trypanosome species were identified in midguts of tsetse flies captured in the Bafia focus.

**Methods:**

Tsetse flies were trapped using pyramidal traps. Each tsetse fly was identified and live flies were dissected and their midguts collected. DNA was extracted from each midgut and thereafter, blood meals and different trypanosome species were identified with molecular tools. The biological data were transported onto maps in order to have their distribution.

**Results:**

Of the 98 traps set up, 461 *Glossina palpalis palpalis* were captured; 322 (69.8 %) tsetse flies were dissected and 49 (15.2 %) teneral flies identified. The average apparent density of tsetse flies per day was 1.18. Of the 35 (10.9 %) blood meals collected, 82 % were taken on pigs and 17.6 % on humans. Eighty two (25.5 %) trypanosome infections were identified: 56 (17.4 %) *T. congolense* savannah, 17 (5.3 %) *T. congolense* forest, 5 (1.6 %) *T. vivax* and 4 (1.2 %) *T. brucei* s.l. No infection of *T. simiae* and *T. b. gambiense* was identified. Sixty seven (81.7 %) infections were single and 15 (18.3 %) mixed involving one triple infection (*T. congolense* forest, *T. brucei* and *T. vivax*) and 14 double infections: 11 *T. congolense* forest and *T. congolense* savannah, two *T. congolense* savannah and *T. brucei*, and one of *T. brucei* and *T. vivax*. The generated maps show the distribution of tsetse flies and trypanosome infections across the focus.

**Conclusion:**

This study has shown that animal trypanosomes remain an important problem in this region. Meanwhile, it is very likely that HAT does not seem anymore to be a public health problem in this focus. The generated maps enabled us to define high risk transmission areas for AAT, and where disease control must be focused in order to improve animal health as well as the quantity of animal proteins.

## Background

Trypanosomiases are infectious diseases affecting humans and animals. They are caused by several trypanosomes species which are most often transmitted by tsetse flies of the genus *Glossina*. African Animal trypanosomiasis (AAT) also known as “nagana” causes major constraints to livestock production in 37 sub-Saharan African countries. About 50 million cattle are at risk of AAT with economic losses estimated at up to US$ 1.3 billion [1]. These economic losses linked to AAT have led to the organization of disease control campaigns both nationally and regionally with initiatives such as the Pan African Tsetse and Trypanosomiasis Eradication Campaign [2].

Human African Trypanosomiasis (HAT) also known as sleeping sickness is an important public health disease in sub-Saharan Africa. On the basis of the HAT-related mortality, it has been ranked ninth out of 25 human infectious and parasitic diseases in Africa [3, 4]. Efforts undertaken in the control of HAT during the last decades brought this disease under control and led to its inclusion in the WHO “roadmap for eradication, elimination and control of neglected tropical diseases”, with a target set to eliminate HAT as a public health problem by 2020 [5]. Beside control activities, studies undertaken to improve our epidemiological knowledge on HAT revealed the presence of *Trypanosoma brucei gambiense* in various domestic and wild animal species of central African HAT foci [6–10]; confirming thus results obtained in West Africa [11–13]. In addition to that, *T. b. gambiense* was often associated with animal trypanosomes in the same tsetse fly as well as the same animal of central African HAT foci [8, 9, 14–16]. In such context where both diseases share the same environment, coexist in the same host and are transmitted by the same tsetse fly species, control measures targeting human and animal diseases can be developed. At this moment where the elimination of HAT is foreseen and where very few cases are detected in most endemic foci, the development of control measures targeting HAT and AAT is becoming more and more important. Whatever the control measures, a deep understanding of the epidemiological context of the disease is required before their establishment. In most active HAT foci, case detection and treatment enable us to update the epidemiological situation of the disease [17]. However, in foci where no control activity has been undertaken for several years such as the Bafia focus, the real epidemiological situation of the disease is unknown. Indeed, for more than 10 years, one passive case of HAT was reported at Mbandjock [17] at about 80 km in the East of Bafia. Following this case, the Bafia-Mbandjock area was considered as at risk (very low level) for HAT [18] and the few active medical surveys undertaken in the whole area revealed no case [17, 19]. The recent update for 2008–2012 period has down-scaled to “marginal”, the risk of HAT in the Bafia focus [17] despite the fact that no medical survey was conducted in this focus for about a decade. The current epidemiological situation of the disease therefore remains vague. Case finding within exposed population is difficult to perform regularly in “silent” foci such as Bafia due to the lack of funds for active surveillances. Similar situations are gradually observed in most foci declaring few cases and where elimination has been foreseen. In such foci, the national sleeping sickness control program is in the process of switching from active case detection by mobile team towards the epidemiological surveillance. Up until now, no reliable appropriate method has been validated for this surveillance. However, in foci where real epidemiological data are not available, the identification of trypanosomes in tsetse flies or xenomonitoring could provide data that could help to understand the current situation of human and animal Trypanosomiases since the transmission of these diseases relies mainly on tsetse flies. Xenomonitoring is gradually becoming an interesting topic in most vector-borne diseases, especially those for which the elimination is foreseen such as lymphatic filariasis [20–22]. The identification of trypanosomes in tsetse flies indicates an active transmission and suggests a potential risk of human infection, thereby pleading in favor to continue with disease control measures. For effective control of African trypanosomiasis, it is important to identify zones with high transmission risk. With the development of Geographical Information System, biological risk factors such as trypanosome infections, teneral flies and blood meals can be integrated onto maps to show their spatial distribution in order to identify areas where the transmission still occurs, and where control must be implemented to achieve the elimination. Up until now, published geo-referenced data on tsetse infection are rare [23], and studies such as the present one will contribute to filling this gap.

In the light of this, the present study was designed to update our knowledge on the current risks of HAT and AAT in the “silent” HAT focus of Bafia of Cameroon by providing entomological data as well as trypanosome infections in tsetse flies captured in villages of this focus. Thereafter, the distribution of tsetse flies and trypanosome infections was performed in order to localize sites and villages where transmission of trypanosomes is still active and where the control operation must be implemented.

### Study zone

**Fig. 1:**
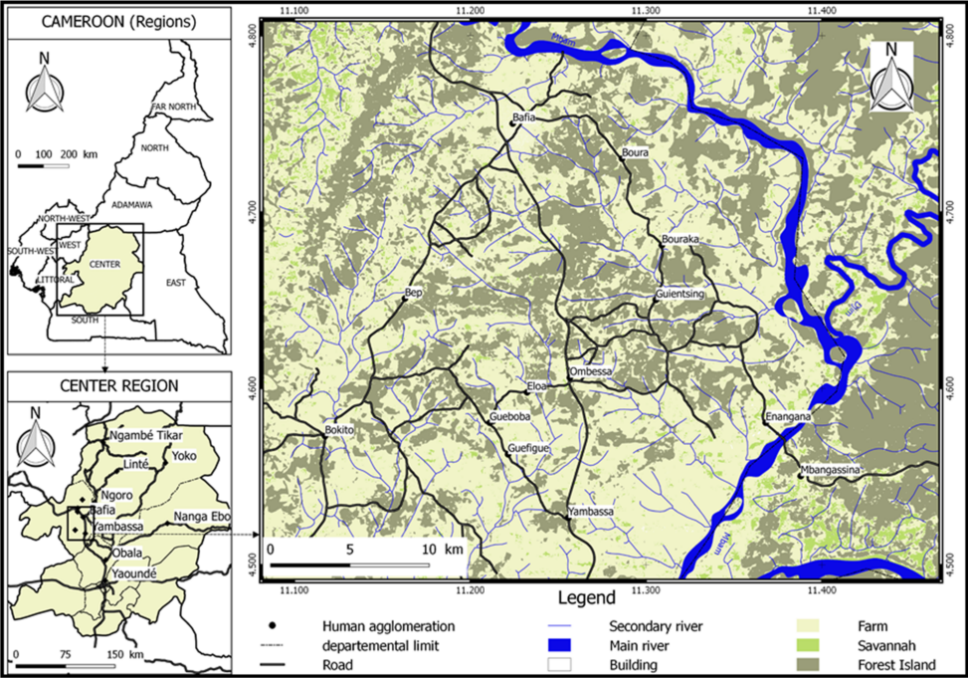
The Bafia sleeping sickness focus showing villages where the entomological surveys were undertaken [20]

This study was conducted in the Bafia HAT focus located in the Mbam-Inoubou District at about 120 km in the northwest of Yaoundé, the political Capital of Cameroon. This HAT focus covers about 1300 km^2^ and was considered as a “silent” focus where no HAT case was reported during the last 20 years despite the case passively reported at Mbandjock, at approximately 80 km from the focus area [18]. The Bafia focus is located in the transition zone between the forest and the savannah. The area has an equatorial type climate with four seasons: two dry seasons (December to February, and July) and two rain seasons (March to June and August to November) [24]. The inhabitants practice peasant agriculture dominated by the cocoa and diverse subsistence crops. They also practice small animal breeding of pigs, sheep and goat. The main ethnic groups include the Bafia, Lemandé and Yambassa. This HAT focus has a dense hydrographic network dominated by the Mbam river (Fig. 1) [25]. It is limited in the north by Bafia, the south by Yambassa, the west by Bokito and the East by Enangana. Until 1990, it was considered as one of the most active HAT areas of Cameroon. Within this focus, villages such as Assala, Guéboba and Guéfigué of the Bokito Sub-districts, Bouraka, Boyaba, Boyabissoumbi, Ningoang and Bogondo of the Ombessa Sub-districts, Doguem and Biabégoura of the Bafia sub-district were the most affected [25]. Amongst these villages, Guéboba, Guéfigué, Bouraka and Ombessa were classified as high-risk due to their localization near the secondary river system [25]. For this study, the entomological surveys were performed in the four villages presented previously as at higher risk (Guéboba, Guéfigué, Bouraka and Ombessa) and one neighboring village (Guientsing) (Fig. 1).

## Methods

### Trapping and dissection of tsetse flies

Two entomological surveys were undertaken in October 2009 and June 2010. During each survey, pyramidal traps [26] were deployed for 4 consecutive days. The geographical coordinates of each trap were recorded using a Global Positioning System. Trapped tsetse flies were collected twice a day. After each collection, tsetse flies were identified morphologically, and then counted and sorted into teneral and non-teneral as described by Laveissière et al*.* [27]. All live flies were dissected in a drop of 0.9 % saline solution using a stereo-microscope. To prevent cross-contamination, the dissecting instruments were carefully cleaned after each dissection by immersing them into a solution of 0.1 M sodium hydroxide and then, in distilled water. The midguts (with and without blood meal) were collected into microtubes containing 95° ethanol. The midguts containing residual blood meals were recorded for subsequent identification of their origins. The microtubes containing midguts were kept at room temperature in the field, and later transferred to −20 °C in the laboratory until use.

### Ethical statement

This study was carried out in strict accordance with the recommendations in the Guide for the Care and Use of Animals of the Department of Biochemistry of the University of Dschang.

### DNA extraction

The microtubes containing midguts were removed from the freezer and incubated in an oven at 80 °C to evaporate ethanol. Thereafter, 300 μl of 5 % chelex [28] were added to each tube. The mixture was homogenized for 10 min at room temperature, and then incubated at 56 °C for 30 min followed by an additional incubation at 98 °C for 30 min. After centrifugation at 13,000 rpm for 10 min, the supernatant containing DNA extract was collected and stored at −20 °C for molecular analyses. For each extraction series, a negative control tube containing only 5 % of Chelex was incorporated and processed at last. This control tube was further used as negative control during PCR reaction in addition to classical PCR negative control consisting of distilled water.

### Identification of different trypanosome species

Trypanosome identification was performed by amplifying the internal transcribed spacer 1 (ITS) of ribosomal DNA as described by Desquesnes et al*.* [29]. The amplification reactions were performed in two PCR rounds as described by Farikou et al*.* [30]. The first round, was performed in a final volume of 20 μl containing 2 μl of DNA extract, 20 picomoles of each primer (TRYP18.2C: 5’-GCAAATTGCCCAATGTCG-3’; TRYP4R: 5’-GCTGCGTTCTTCAACGAA-3’), 200 mM of each dNTP and 0.5 unit of Taq DNA polymerase. The amplification program began with a denaturation step at 94 °C for 3 min followed by 30 amplification cycles; each cycle containing a denaturation step at 94 °C for 30 s, an annealing step at 51 °C for 30 s, and an extension step at 72 °C for 2 min followed by a final extension at 72 °C for 10 min. The amplified products were diluted 10 fold and 2 μl of each dilution was used as template for the nested PCR. This latter was performed with IRFCC (5’CCTGCAGCTGGATCAT 3’) and TRYP5RCG (5’ATCGCGACACGTTGTG 3’) primers. The amplification program was identical to the one described for the first PCR.

After the nested PCR, 10 μl of amplified products were resolved on 2 % agarose gel which was subsequently stained with ethidium bromide. The gels were visualized on ultraviolet light and photographed. At this step, *T. brucei* s.l., *T. vivax* and *T. congolense* can easily be identified. However, for different types of *T. congolense* as well as *T. brucei* subspecies, specific primers were subsequently used.

### Identification of different types of *T. congolense*

*T. congolense* subtype identification was performed on all samples that showed a DNA fragment between 600 bp and 650 bp corresponding to the expected size of *T. congolense* species. For this identification, specific primers for *T. congolense* forest and savannah [31] were used to amplify specific DNA sequences of each of them. PCR reactions were performed as described by Simo et al. [15]. The amplified products were analyzed on 1.5 % agarose gel which was subsequently stained with ethidium bromide and visualized under UV light.

### Search for *T. b. gambiense*

This was done only on samples that showed a DNA fragment of 392 bp corresponding to the expected size of trypanosomes of the subgenus *Trypanozoon (T. b. brucei, T. evansi, T. b. gambiense* group 1 and *2, T. b. rhodesiense)*. For these samples, a second PCR was performed as described by Simo et al. [8] using TRBPA1/2 primers that amplify an allele of 149 bp characteristic of group 1 *T. b. gambiense*. All samples that did not show the 149 bp allele amplicon following the second PCR were considered as being infected by *T. b. brucei,* since the other species (*T. evansi* and *T. b. rhodesiense*) of the subgenus *Trypanozoon* are probably absent in the region due to their geographical localization.

### Identification of tsetse fly blood meal origin

The origin of tsetse blood meals was determined using the heteroduplex PCR-based assay as described by Njiokou et al. [32]. Briefly, Cytochrome B gene was amplified using Cytochrome B primers [33]. The amplification program was as follows: a denaturation step at 95 °C for 3.5 min and 40 amplification cycles; each cycle having a denaturation step at 95 °C for 30s, an annealing step at 58 °C for 50s and an extension step at 72 °C for 1 min followed by a final extension at 72 °C for 5 min. Heteroduplexes were formed by hybridization of each amplified cytochrome B gene with that of Giant rat (*Crycetomys gambianus*) used as a driver. The heteroduplex profiles were resolved on 5 % acrylamide/urea gels and blood meal origin was identified by comparing the heteroduplex profile of each blood meal with those of vertebrates (man, pig, goat, sheep and dog) used as references.

To visualize the distribution of traps, tsetse flies density and trypanosome infections on a map, two sources of data have been used: the digital globe satellite image (30 cm of spatial resolution) downloaded from https://www.bing.com/maps/, and Radar image (SRTM, 1 arc second) downloaded from http://earthexplorer.usgs.gov. The first image was use to draw the land use map by supervised classification and interpretation (digitalization of Main River, road and building) and the second image enabled us to extract hydrographic network under Grass Gis software version 6.4. The land use map was generated out of supervised classification combined with interpretation (digitalization of roads, Mbam river and Building) of Digital globe image downloaded from https://www.bing.com/maps/ previously geo-referenced. The hydrographic network was extracted from SRTM (1 arc second) downloaded freely from http://earthexplorer.usgs.gov/.

Geographical coordinates of each trap and related information (Id of Tsetse fly trap, description of the station, number of captured tsetse fly and, their state) contained in an attribute table were projected on land use map using Qgis software and the thematic analysis was carried out at the level of traps in order to evaluate the distribution of tsetse flies and different trypanosome species.

### Data analysis

The apparent density of tsetse flies per trap per day (ADT) was calculated to assess the relative abundance of tsetse flies at each trapping site using the formula below.

ADT = C/TD; where C is the number of tsetse flies caught, T the number of traps deployed and D the number of days of trapping.

Statistical analyses enabled to compare data between villages. The ANOVA test for paired-samples was used to compare the ADTs between villages. The Pearson chi-square test was used to compare the percentages of dissected and teneral tsetse flies as well as tsetse flies infected by at least one trypanosome species. The Fisher's exact test was used to compare the infection rates of different trypanosome species as well as the proportion of blood meals. Yates correction for continuity or Fisher's exact test was used for samples of small size. The threshold for significance was set at 5 %.

## Results

### Entomological surveys

**Fig. 2:**
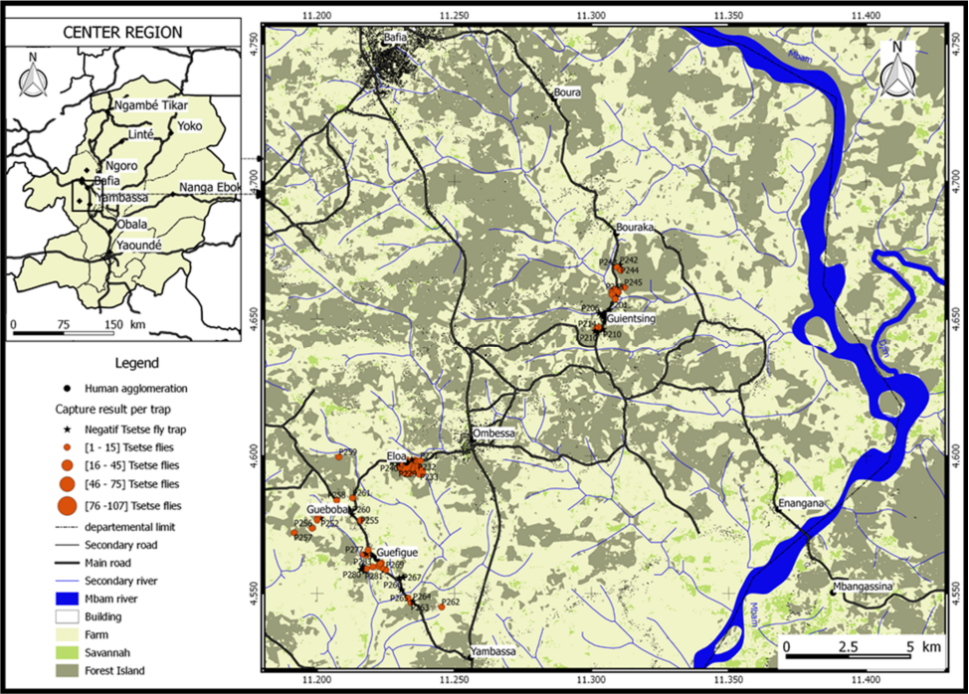
Map showing the distribution of traps as well as the relative number of tsetse flies captured at each trap

For this study, 98 pyramidal traps were set up in five villages (Fig. 2): 40 at Eloa, 16 at Guientsing, 10 at Bouraka, 22 at Guefigue and 10 at Gueboba. For the 98 traps, 461 tsetse flies were captured: 226 (49.02 %) during the first survey and 235 (50.98 %) in the second. All tsetse flies captured were of the genus *G. p. palpalis*. The ADPs varied between traps and villages with an average of 1.18 tsetse flies per trap per day (Table 1). The highest ADT of 1.92 was obtained at Eloa and the lowest of 0.06 at Guientsing. At trap level, the ADPs varied from zero to 12 (Fig. 2). Significant differences (*P* < 0.000) were obtained by comparing ADPs between villages (Table 1).

**Table 1:** Results of entomological surveys

Villages	Captured flies	ADP	Dissected flies (%)	Teneral flies (%)	Blood meals (%)
Eloa	307	1.92	223 (72.3)	25 (11.2)	30 (13.4)
Bouraka	37	0.93	24 (73)	2 (8.3)	0 (0)
Gueboba	72	1.80	36 (50)	6 (16.7)	5 (13.9)
Guefigue	41	0.47	35 (85.4)	4 (11.4)	0 (0)
Guientsing	4	0.06	4 (100)	2 (50)	0 (0)
Total	461	1.18	322 (69.8)	49 (15.2)	35 (10.9)
*P* value		<0.001	<0.01	<0.001	0.045

*ADP* apparent density of tsetse flies per trap per day

Out of 461 tsetse flies captured, 69.8 % (322/461) of them were dissected and 49 (15.2 %) teneral flies were identified. The percentage of teneral flies varied between villages and capture sites (Table 1). The lowest percentage of teneral flies was observed at Bouraka (8.3 %) and the highest at Guientsing (50 %). However, the sample size was very low at Guientsing since only four tsetse flies were captured. A significant difference (*X*^*2*^ = 27.023*; P < 0.000*) was observed when the percentages of teneral flies were compared between villages.

### Blood meal identification

Thirty five (10.9 %) blood meals were collected: 28 (80 %) during the first survey and 7 (20 %) in the second. No blood meal was collected in tsetse flies captured at Bouraka, Guefigue and Guientsing. In contrast, 30 and 5 blood meals were collected in flies captured at Eloa and Gueboba, respectively. A significant difference (*P < 0.05*) was observed in the proportion of blood meals between villages (Table 1). Blood meal analysis shows that 82.4 % were taken on pigs and 17.6 % on humans. However, variations were observed between entomological surveys. For instance, 92.3 % of blood meals were taken on pigs (7.7 % on humans) during the first survey and 50 % in the second (50 % on humans).

### Molecular identification of trypanosomes

Of the 322 tsetse flies that were dissected and whose midguts were subjected to the molecular identification of trypanosomes, 25.5 % (82/322) of them were infected by at least one trypanosome species: 56 (17.4 %) *T. congolense* savannah, 17 (5.3 %) *T. congolense* forest, 5 (1.6 %) *T. vivax* and 4 (1.2 %) *T. brucei* s.l. No infection of *T. simiae* was identified. *T. congolense* forest and savannah were the main trypanosome species found in tsetse flies of the Bafia focus. Amongst the four *T. brucei* s.l. infections, no infection due to *T. b. gambiense* was detected; suggesting thus the absence of human pathogenic trypanosome in tsetse flies of the five villages. Irrespective of the village, the infection rate of *T. congolense* savannah was always higher (about 6 times at Gueboba) than those of *T. brucei* or *T. vivax*. At Gueboba, the infection rate of *T. congolense* forest was higher than that of *T. congolense* savannah. In the other villages, the infection rate of *T. congolense* savannah was higher than that of *T. congolense* forest. Five infections of *T. vivax* were identified in tsetse flies of two villages: one infection at Gueboba and four infections at Guefigue. Four *T. brucei* infections were identified in tsetse flies of three villages: two tsetse of Guefigue, one of Bouraka and one of Gueboba. No trypanosome infection was recorded at Guientsing where only 4 tsetse flies were captured. The highest trypanosome infectious rate of 54.3 % (19/35) was recorded at Guefigue followed by 41.7 % (15/36) at Gueboba, 20.8 % (5/24) at Bouraka and 19.3 % (43/223) at Eloa (Table 2). *T. congolense* (savannah and forest) was the only trypanosome species identified in tsetse flies captured at Eloa. *T. congolense* and *T. brucei* were identified in tsetse flies of Bouraka whereas *T. congolense*, *T. brucei* and *T. vivax* were found in tsetse flies of Gueboba and Guefigue. Comparing the trypanosome infection rates between villages, significant differences were observed for *T. congolense* forest (*P = 0.001*), *T. brucei* s.l. (*P = 0.01*) and *T. vivax* (*P < 0.001*). However, no significant difference was observed (*P = 0.437*) for *T. congolense* savannah (Table 2).

**Table 2:** Results of the molecular identification of trypanosomes

		PCR results				
Villages	Dissected flies (%)	TCF (%)	TCS (%)	TB (%)	TVW (%)	NTI (%)
Eloa	223 (72.3)	4 (1.8)	39 (17.5)	0 (0)	0 (0)	43 (19.3)
Bouraka	24 (73)	1 (4.2)	3 (12.5)	1 (4.2)	0 (0)	5 (20.8)
Gueboba	36 (50)	7 (19.4)	6 (16.7)	1 (2.8)	1 (2.8)	15 (41.7)
Guefigue	35 (85.4)	5 (14.3)	8 (22.9)	2 (5.7)	4 (11.4)	19 (54.3)
Guientsing	4 (100)	0 (0)	0 (0)	0 (0)	0 (0)	0 (0)
Total	322 (69.8)	17 (5.3)	56 (17.4)	4 (1.2)	5 (1.6)	82 (25.5)
VFET	21.108^a^	18.43	3.5	12.58	18.32	21.11
*P* value	<0.001	0.001	0.437*	0.01	0.001	0.001

*VFET* value of Fisher's Exact Test, *TCF Trypanosoma congolense* forest, *TCS Trypanosoma congolense* savannah, *TB Trypanosoma brucei* s.l., *TVW Trypanosoma vivax*, *NTI* number of tsetse flies infected by at least one trypanosome species

*: *P* value not significant; ^a^: Pearson Chi-Square

Of the 82 tsetse flies infected by at least one trypanosome species, 81.7 % (67/82) of them were single infections and 18.3 % (15/82) mixed infections of different trypanosome species. Of these 15 mixed infections, one of them was a triple infection of *T. congolense* forest, *T. brucei* and *T. vivax*. The 14 remaining include 11 double infections of *T. congolense* forest and *T. congolense* savannah, two of *T. congolense* savannah and *T. b. brucei*, and one of *T. b. brucei* and *T. vivax*. The mixed infections were identified in all villages where at least one trypanosome has been reported: 6 mixed infections at Gueboba, 5 at Guefigue, 3 at Eloa and one at Bouraka.

**Fig. 3:**
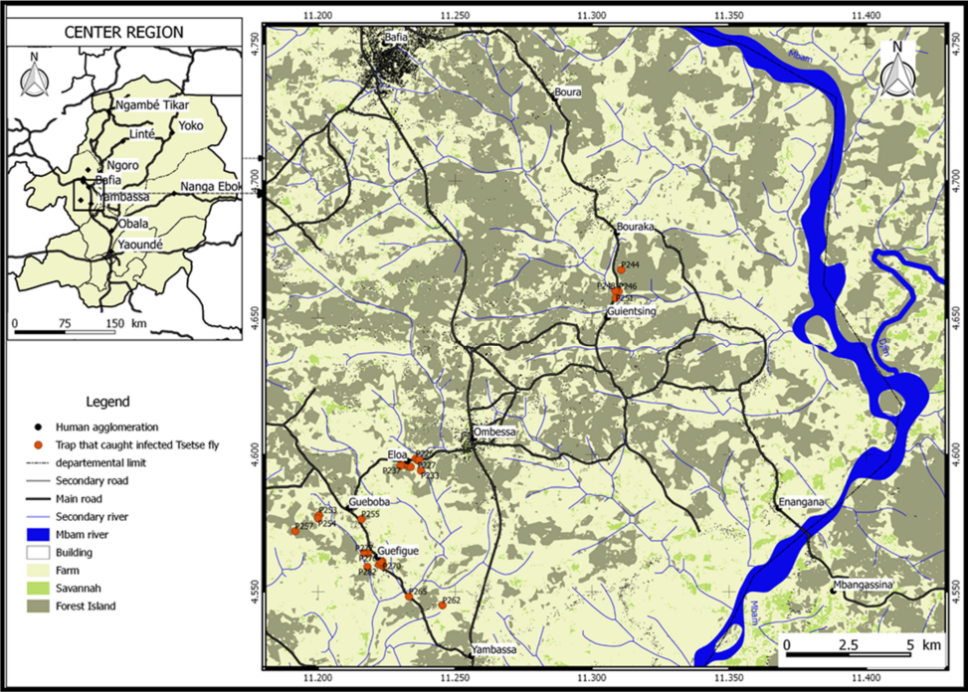
Map showing the distribution of traps that have captured tsetse flies which shows at least one of them infected by trypanosomes

Figure 2 shows that 51 (52 %) out of 98 traps that were set up have captured at least one tsetse fly. The number of captured flies varies from zero to more than one hundred (Fig. 2). Amongst the traps deployed, 27 (27.6 %) of them captured at least one infected tsetse fly (Fig. 3). Although the infected flies were widely distributed in different villages, most of these flies were found in neighboring traps (Fig. 3).

## Discussion

During the last decades, considerable efforts were undertaken to map the distribution of tsetse flies as well as their infection rates in order to address the problem linked to trypanosomiasis transmitted by tsetse flies at the continental level [34]. For such initiative and in addition to trypanosome infections in vertebrate hosts, particular attention was given to infections in tsetse flies, although these types of datasets are scant. Geo-referenced data on trypanosome infections in tsetse flies are important data that complement those of AAT and HAT in order to estimate the disease risks for a broader perspective. The present study is within the framework of this continental initiative due to data provided on tsetse fly distribution as well as trypanosome infections in these vectors. The identification of *G. p. palpalis* as the main biological vector of Trypanosomiasis in this region confirms results obtained in the same villages more than 17 years ago [35, 36]. These results indicate that despite the environmental modifications that occurred during the last decades, there are still favorable biotopes for tsetse flies. Significant differences were observed for the ADPs between villages. Additionally, significant differences were also observed between the mean of ADPs (1.15) in this study and the mean value of 6.9 reported 17 years ago in the same region [36]. The differences observed between villages and sampling periods could be linked to different anthropization levels that can be observed between villages, but also that occurred between trapping periods. Indeed, the environmental and socio-economical mutations that occurred have induced the development of farm lands through deforestation. These human activities have considerably modified tsetse biotopes which have had direct effects on the fly density.

The 10.9 % of residual blood meals obtained here is considerably higher than the 2.8 and 4.7 % obtained for the same tsetse species captured in the forest foci of southern Cameroon [30]. These differences could be explained by the fauna composition as well as the number of domestic and wild animal that can be found in each focus. Indeed, in the HAT focus of the forest regions of southern Cameroon, different wild animal species are found [9, 14] and very few domestic animals are bred. In such context, tsetse flies find difficulties to take their blood meals due to the high vivacity of wild animals compared to domestic animals. In the Bafia HAT focus where the environment is highly anthropized, wild animals are rare and consequently, most blood meals are taken on humans or domestic animals. This hypothesis is strengthened by our results showing that 82.4 % of blood meals were taken on pigs. Humans appeared as the second host on whom tsetse flies took their blood meals. However, the nutritional behavior of tsetse flies can change rapidly with time, as was observed in our study where 92.3 % of blood meals were taken on pigs during the first survey and only 50 % in the second survey. These results are in agreement with those reported in HAT foci of forest regions of southern Cameroon [30]. Such modifications will impact the transmission of parasites and therefore, must be taken into account during the development and establishment of control measures. The treatment of infected animals associated to vector control in villages and zones showing high disease transmission risk will enable the reduction of disease incidence.

The different trypanosome species identified in this study are in agreement with results reported in tsetse flies of other African regions [15, 31–35]. All trypanosome species identified in this study were reported in tsetse flies of the same villages about 17 years ago [36]; showing that the transmission of the same parasites continues to occur despite the environmental and socio-economical mutations that occurred in the region. Our results suggest that trypanosome infections probably remain a serious threat for animal breeding in this zone. They revealed that 25.5 % of tsetse flies were infected by at least one trypanosome species. This percentage is higher than 19 % previously obtained by Morlais et al. [36] in the same HAT focus. If we take into consideration the fact the investigations were performed only on tsetse midguts, we can therefore infer that the prevalence reported here was underestimated since the trypanosomes infecting the proboscis and salivary glands (mature infections of trypanosome species such as *T. brucei*) were not considered in the present study. Despite this underestimation, the increase in prevalence with time indicates that trypanosome transmission is becoming more and more important despite the reduction of tsetse fly density. The high prevalence of tsetse infected by animal trypanosomes can be linked to blood meal analyses showing that more than 80 % of the blood meals were taken on animals. In such context, there is a high probability of tsetse flies to get infected by animal trypanosomes. These results strengthen the fact that AAT may constitute a serious threat that needs to be considered for actions aiming to improve the animal production and peasant economy.

The single and mixed infections identified in this study confirm results obtained in tsetse flies and mammals of different African countries [16, 37–41]. These results show that, whatever the region, single and mixed infections of different trypanosome species are frequent in tsetse flies. The mixed infections reflect probably their prevalence in mammals, or the frequent contacts between tsetse flies and animals infected by different trypanosome species. Despite the similar profiles (single and mixed) of infections observed across sub-Saharan African Countries, the percentage of mixed infections can vary between countries, and even within regions of the same country. For instance, 15 % of mixed infections were observed in this study whereas only 7.02 % were reported for the same tsetse species in the Malanga focus in Democratic Republic of Congo [15]. In Côte d’Ivoire, mixed infections were reported in 25 % [37], 40 % [40] and 64 % [41] of tsetse flies. For the same region, the percentage of mixed infections can change with time like in the Bafia focus where 40.43 % of these infections were reported 17 years ago [36] and only 15 % in this study. These results show that the types of infections (simple or mixed) can vary significantly between regions and also with time. Until now, the factors determining the distribution and the abundance of trypanosomes in a particular region are poorly known. It is likely that most of these factors include the availability of suitable animal species as well as the suitable tsetse fly species to ensure the transmission of specific trypanosome species.

Amongst the trypanosome species identified in this region, *T. congolense* was the most common species, and *T. congolense* savannah the most prevalent. These results corroborate those reported in tsetse flies of West [37, 38, 40, 41], Central [15, 36] and East Africa [39]. The high prevalence of *T. congolense* savannah can be explained by the geographical localization of the study area. The Bafia HAT focus is located in the transition zone where the vegetation is similar to that found in the savannah zone. The high prevalence of *T. congolense* confirms the results of Morlais et al. [36] reporting, in the same region, more than 2 times *T. congolense* than *T. brucei*. The high prevalence of *T. congolense* corroborates results obtained in West Africa where similar profiles of infections were observed both in tsetse flies than animals of the same villages [38]. Assuming that high infection rates of *T. congolense* savannah can also be observed in animals of our study zone, it is likely that AAT must have real impacts on the animal health in the Bafia focus. Knowing the pathological impacts of trypanosome infections, it is obvious that the high infection rates of these parasites will impact the peasant economy by affecting animal breeding in terms of animal health, productivity, quality and quantity of meat. With the high pathogenic effects of *T. congolense* and especially the savannah type, its high prevalence constitutes an additional element strengthening the need to control AAT in the Bafia focus. The maps (Figs. 2 and 3) presenting the distribution of tsetse flies and infected flies allow for the identification of villages and areas at high transmission risk, and where control must be targeted. This distribution is very important for the planning of a control operation because the prevalence and the transmission of the disease can differ according to villages. Approaches defining areas for targeted control activities will lead not only to a reduction of control costs, but also to an enhancement of efficacy.

The identification of *T. vivax* in the midgut of tsetse flies is surprising because the life cycle of this parasite was described as confined to proboscis. Nevertheless, it has been reported that the development of *T. vivax* seems to be initiated in the oesophageal region of tsetse flies [42]. Our results are in agreement with those of previous studies [15, 16, 36] reporting *T. vivax* DNA in the midguts of tsetse flies with molecular tools. It is likely that some tsetse flies can take their blood meals on mammals infected by *T. vivax,* and after partial digestion, residual DNA of this parasite can be detected by PCR.

The identification of tsetse flies infected by *T. brucei* s.l. confirms previous results [36]. However, the low prevalence (1.2 %) reported here does not corroborate the 7.08 % obtained 17 years ago in the same region, and also the 4.7 and 3 % reported in the HAT foci of Fontem and Bipindi, respectively [16, 43]. The low prevalence observed in this study could be explained by the fact that some midgut infections of active foci are due to *T. b. gambiense*. This hypothesis is strengthened by the absence of detection of *T. b. gambiense* in tsetse flies of the Bafia focus and its detection in flies of active foci such as Fontem and Bipindi [16, 43]. This absence of detection of *T. b. gambiense* is in agreement with epidemiological data reporting no case in the Bafia focus in the past 20 years despite medical surveys and one case passively detected at Mbandjock, a neighboring area located at about 80 km in the East of this focus [18]. The complete absence of reported cases in the Bafia focus during the period of 2008–2012 has down-scaled to “marginal”, the risk of the disease in this focus [17]. Though animals have not been investigated for *T. b. gambiense* infections, all data generated until now play in favor of the “silent” status of the Bafia focus.

The very low prevalence of *T. b. brucei* could be explained by the fact that wild animals that are known as being the reservoir of these parasites as well as the source of new infections are scarce due to the environment that has been subjected to high anthropization. Such an observation has already been reported in HAT focus of Malanga in Democratic republic of Congo [15], indicating thus a very low transmission rate of *T. b. brucei*.

## Conclusion

This study provided indirectly an update on the current risks of human and animal trypanosomiases in the Bafia focus. It also provided data that enabled us to partially fill the gap observed on the published geo-referenced data on tsetse infections. The results obtained suggest that animal trypanosomes remain an important problem that needs to be considered in this region. The absence of detection of *T. b. gambiense* in tsetse flies indicates that HAT does not seem to be a public health problem in this focus anymore. The maps generated in this study enabled us to localize areas showing high risk for AAT and where disease control must be focused in order to improve animal health. The establishment of control activities in these areas will help to sustain peasant economy, and will also supply animal proteins for inhabitants to whom hunting is prohibited due to protected animal species.
